# Bethanechol-associated hyperinsulinemic hypoglycemia in a child with autonomic instability

**DOI:** 10.1210/jcemcr/luag294

**Published:** 2026-10-05

**Authors:** Rashna Soonavala, Apisadaporn Thambundit, Andrew Kanouse

**Affiliations:** Department of Pediatrics, David Geffen School of Medicine at University of California Los Angeles, Los Angeles, CA 90095, USA; Division of Endocrinology, Department of Pediatrics, University of California Los Angeles, Los Angeles, CA 90095, USA; Division of Endocrinology, Department of Pediatrics, University of California Los Angeles, Los Angeles, CA 90095, USA

**Keywords:** hyperinsulinism, bethanechol, cholinergic agonist, hypoglycemia, autonomic instability

## Abstract

Hyperinsulinemic hypoglycemia (HH) is a rare but potentially dangerous condition characterized by inappropriate insulin secretion. This case describes a pediatric patient with HH in the setting of autonomic instability following neurologic injury. He was started on bethanechol, a muscarinic receptor agonist, for gastric dysmotility and developed severe, persistent hypoglycemia shortly after a dose increase with laboratory findings confirming dysregulated insulin secretion. The hypoglycemia was difficult to stabilize, requiring high glucose-infusion rates. Discontinuation of bethanechol was followed by gradual resolution. This temporal association raises the possibility that cholinergic stimulation contributed to dysregulated insulin secretion in a susceptible physiologic state. Muscarinic M3 receptors on pancreatic β-cells enhance insulin secretion, although dysregulation of this pathway by M3 agonists has primarily been demonstrated in murine models and has not previously been reported in humans. This case highlights a potential link between parasympathetic signaling and insulin regulation in autonomic instability and suggests that cholinergic agents may contribute to dysregulated glucose homeostasis in susceptible patients.

## Introduction

Hyperinsulinism is inappropriate insulin secretion causing hypoglycemia. Causes include genetic variants, medications, insulinomas, and central nervous system dysregulation [1]. Hyperinsulinism is most common in neonates due to genetic variants (eg, *ABCC8* or *KCNJ11*) [2]. Bethanechol is a muscarinic (M3) cholinergic agonist used to treat gastrointestinal and genitourinary dysmotility [3]. In murine models, cholinergic signaling influences insulin regulation via pancreatic β-cell M3 receptor activation [4]. Bethanechol is used in children with autonomic instability to treat urinary retention and gastric dysmotility.

Autonomic instability describes dysregulation of the sympathetic and parasympathetic nervous systems, often seen in pediatric patients with spinal cord dysfunction [5]. Changes in autonomic tone can affect β-cell pancreatic function, as increased vagal activity enhances insulin secretion via muscarinic stimulation [6]. Individuals with spinal cord lesions may be at increased risk for insulin dysregulation due to disruption of autonomic pathways involved in glucose regulation [7].

This case describes a 5-year-old boy with new-onset paraplegia and autonomic instability secondary to a posterior fossa medullary abscess. He was treated with bethanechol for gastrointestinal dysmotility and reflux, and then developed hyperinsulinemic hypoglycemia (HH) shortly after a dose increase. Hypoglycemia resolved after discontinuation, raising the possibility that bethanechol contributed to HH in the setting of autonomic instability.

Although bethanechol alters insulin and glucose regulation in animal models, this association has not previously been demonstrated in humans [8]. This case highlights a potential association in a vulnerable population and the need for clinical awareness and monitoring.

## Case presentation

A previously healthy 5-year-old boy presented to the emergency department in respiratory arrest with acute loss of extremity motor function. He was emergently intubated and started on empiric antibiotic treatment for presumed meningitis with cefepime and metronidazole. Subsequent evaluation included a posterior fossa craniotomy and C1 laminectomy that revealed a medullary/fourth-ventricle abscess with fungal elements. He was treated with liposomal amphotericin B, micafungin, ceftazidime, ceftriaxone, and fluconazole.

His initial hospitalization was complicated by resultant spastic quadriplegia, ventilator- and gastrostomy tube (G-tube) dependence, and recurrent asystolic episodes requiring a transvenous pacemaker. He was discharged home after 2.5 months.

He returned 1.5 weeks following discharge with 10 days of fever, an increased oxygen requirement, and worsening respiratory secretions. He tested positive for rhinovirus/enterovirus and was admitted for supportive care. Tracheal aspirate cultures later grew *Pseudomonas aeruginosa*, *Enterobacter cloacae* complex, *Staphylococcus aureus*, and *Chryseobacterium indologenes*. At the start of this admission, he was receiving home medications of albuterol, pulmozyme, budesonide, fluconazole, gabapentin, melatonin, trazodone as needed (PRN), baclofen, buspirone PRN, lorazepam PRN, and simethicone. He received urinary catheterization PRN for retention with initially normal creatinine. Hepatic function upon presentation was normal.

He was started on bethanechol for gastroesophageal reflux and gastrointestinal dysmotility. He later developed persistent abdominal bloating, so his bethanechol dose was increased from 0.1 mg/kg 3 times daily to 0.2 mg/kg 4 times daily. The day following this increase—2 weeks into admission—he developed an acute altered mental status with rapid eye blinking, lip pursing, decreased responsiveness, and fixed, dilated pupils with leftward deviation. He was also hypothermic (34.7 °C; 94.5 °F). A point-of-care (POC) glucose was obtained that was found to be undetectable (<10 mg/dL; 0.6 mmol/L-reference 60-100 mg/dL (3.3-5.5 mmol/L)) twice. He received a bolus of dextrose-containing fluids and was started on continuous intravenous (IV) dextrose fluids at 100 mL/hour for a glucose-infusion rate (GIR) of 9.3 mg/kg/minute. POC glucose rose to 152 mg/dL (8.44 mmol/L). Glucose increased to 376 mg/dL (20.87 mmol/L), prompting weaning and stopping of IV fluids. His POC glucose dropped to 29 mg/dL (1.61 mmol/L) about 3 hours after the IV fluids were discontinued, resulting in another dextrose-10% containing (D10) bolus, after which D10 was restarted at 50 mL/hour (4.6 mg/kg/minute). POC glucoses fluctuated from 271 mg/dL (15.04 mmol/L) to 41 mg/dL (2.28 mmol/L), requiring escalation of IV fluids to 60 mL/hour (5.6 mg/kg/minute). Overnight, his lowest blood glucose was 75 mg/dL (4.16 mmol/L) and ultimately the maximum rate he required of D10-containing fluids was 100 mL/hour (9.3 mg/kg/minute). The following day, POC glucoses ranged from 99 to 163 mg/dL (5.49-9.05 mmol/L) until the evening, when his POC glucose dropped to 55 mg/dL (3.05 mmol/L) following a decrease in his fluids to 20 mL/hour (1.9 mg/kg/minute). Three days following his initial hypoglycemia, POC glucoses were stable. G-tube feeds were extended, allowing gradual weaning of his GIR. Bethanechol was discontinued the day of the first hypoglycemic event.

A computed tomography scan following the initial hypoglycemia episode showed no acute intracranial bleed and confirmed severe spinal cord atrophy. He had further seizure-like activity during this time for which he received 3 doses of lorazepam and a levetiracetam bolus. He also had a heart rate of 46 beats/minute, triggering his pacemaker. An electroencephalogram did not reveal epileptiform discharges that would suggest seizure activity.

Altogether, these episodes were thought to be consistent with autonomic instability related to medullary injury. Of note, his feeds had briefly been held 2 days prior due to abdominal pain but were resumed the day of/the day before the episode. GIR was weaned as able to maintain glucose levels between 100 and 160 mg/dL (5.55-8.88 mmol/L).

Because of the concern regarding cerebral abnormalities with hypoglycemia, a pituitary workup was undertaken that was normal: thyroid-stimulating hormone 5.5 μIU/mL (reference, 0.56-5.42 μIU/mL), free thyroxine 1.25 ng/dL (16 pmol/L; reference, 1.08-1.93 ng/dL [13.9-24.8 pmol/L]), and total cortisol 6 μg/dL (165.5 nmol/L; reference, >5 μg/dL [>137.9 nmol/L]).

## Diagnostic assessment

Over the next 5 days, he had intermittent hypoglycemia (to the 60s mg/dL (around 3.3 mmol/L)) requiring dextrose-containing fluid boluses. Five days after the initial episode, he again became hypoglycemic with a POC glucose of 43 mg/dL (2.4 mmol/L). Critical labs at that time showed: serum glucose 45 mg/dL (2.5 mmol/L), total cortisol 55 μg/dL (1517 nmol/L), β-hydroxybutyrate 0.8 mg/dL (0.08 mmol/L), total growth hormone 21.2 ng/mL, and total insulin 74 µIU/mL (514 pmol/L). See normal reference ranges below. A 0.5 mg IV glucagon challenge was performed that increased his POC glucose to 80 mg/dL (4.4 mmol/L) at 20 minutes and 114 mg/dL (6.3 mmol/L) at 30 minutes, consistent with hyperinsulinism (Table 1). Abdominal ultrasound and magnetic resonance imaging showed no evidence of an insulinoma.

**Table 1:** Laboratory evaluation during time of hypoglycemia

Parameter	Patient value	Reference range during hypoglycemia [9]	Clinical interpretation
Serum glucose	45 mg/dL (2.5 mmol/L)	>50 mg/dL (2.78 mmol/L)	Confirmed hypoglycemia
Serum insulin	74 µIU/mL (514 pmol/L)	Undetectable by assay	Inappropriately elevated
β-Hydroxybutyrate	0.8 mg/dL (0.08 mmol/L)	>18.7 mg/dL (1.8 mmol/L)	Suppressed ketosis
Cortisol	55 µg/dL (1517 nmol/L)	>18 μg/dL during stress (497 nmol/L)	Appropriate stress response
Growth hormone	21.2 ng/mL	>5 ng/mL	Appropriate stress response
Glucagon challenge	+69 mg/dL (+3.8 mmol/L)	≥30 mg/dL (1.67 mmol/L) rise in glucose	Diagnostic of hyperinsulinism

## Treatment

Given the persistent HH, he was started on cornstarch 1 g/kg with each of his 4 times daily (QID) feeds and free-water boluses. He was hospitalized for 1 month and discharged on G-tube feeds (Alfamino 300 mL QID), free water (100 mL QID), cornstarch supplementation (1 g/kg QID), and home glucose monitoring. Endocrine input 2.5 weeks after the initial episode notes his lowest glucose to be 79 mg/dL (4.38 mmol/L).

## Outcome and follow-up

One month later, feeds were transitioned to gastrojejunal (GJ) due to G-tube intolerance. He was readmitted for pneumonia 3 months later, during which glucose levels remained stable. By his endocrine follow-up at 4.5 months, he was tolerating 18-hour GJ feeds at 66 mL/hour and cornstarch had been discontinued due to concern for *Clostridioides difficile* infection. Glucoses were between 70 and 100 mg/dL (3.9-5.6 mmol/L) without cornstarch.

At an outpatient endocrinology visit 18 months later, his glucose levels remained stable. The family was performing glucose evaluations if feeds were paused >6 hours; the lowest documented value had been 70 mg/dL (3.9 mmol/L).

See Fig. 1 for a timeline of events.

**Figure 1:**
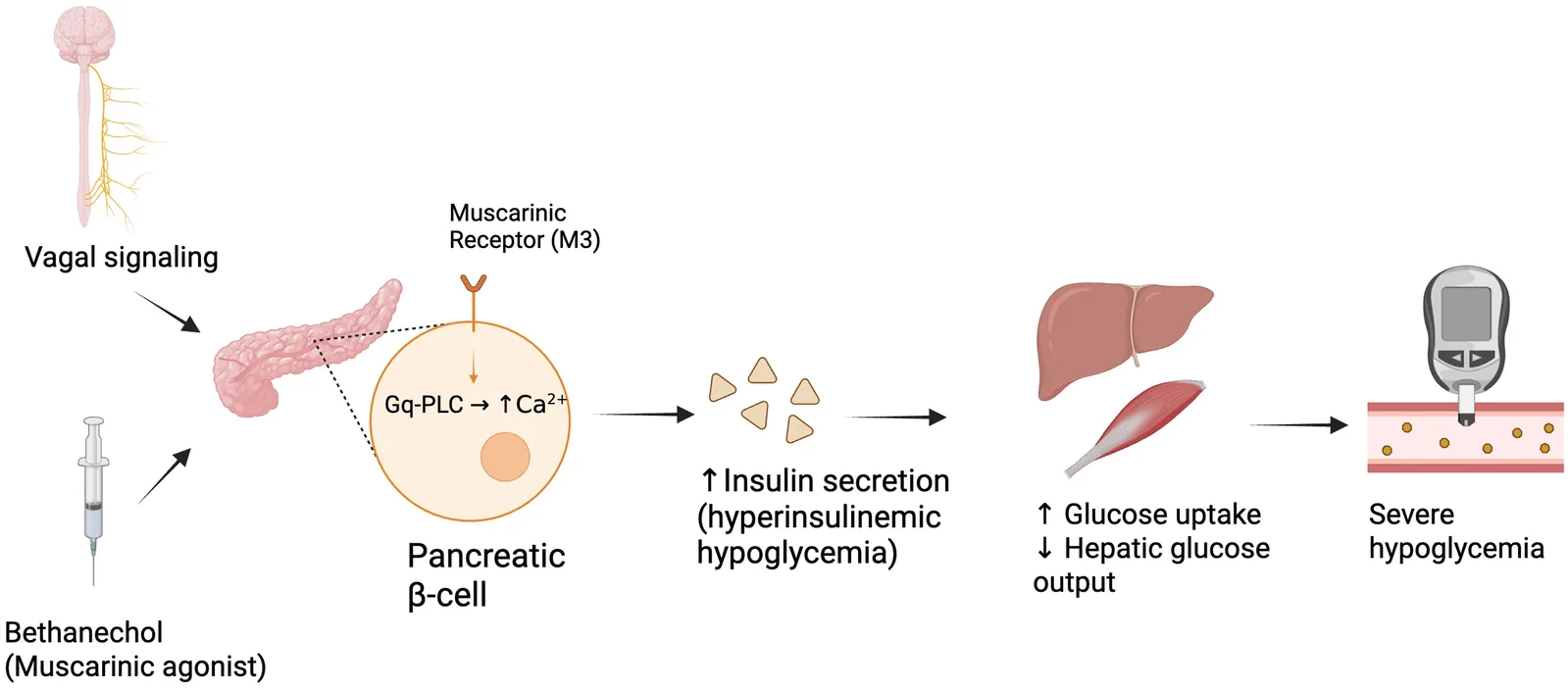
Hypothesized mechanism of bethanechol-associated hyperinsulinemic hypoglycemia in autonomic instability. Created in BioRender. Soonavala, R. (2026). https://BioRender.com/4vean39.

## Discussion

This case describes persistent HH in a 5-year-old boy with a medullary abscess and autonomic instability that developed shortly after an increase in bethanechol dosing. Findings were consistent with insulin-mediated hypoglycemia, including elevated insulin, suppressed ketones, and a strong glycemic response to glucagon (>60 mg/dL rise). Imaging excluded structural causes and the patient had no prior history of hypoglycemia. The temporal relationship with bethanechol dose escalation is consistent with a potential contribution of cholinergic-mediated insulin dysregulation.

Pancreatic β-cells express muscarinic acetylcholine receptors, with the M3 subtype mediating cholinergic insulin secretion [10]. M3 receptors couple with Gq proteins, activating phospholipase C to generate diacylglycerol and inositol triphosphate, increasing intracellular calcium and promoting insulin exocytosis [6]. Experimental models show that impaired M3 signaling reduces insulin secretion, whereas overexpression enhances it [4, 11]. As a direct muscarinic agonist, bethanechol may activate this pathway, contributing to the observed HH (Fig. 2).

**Figure 2:**
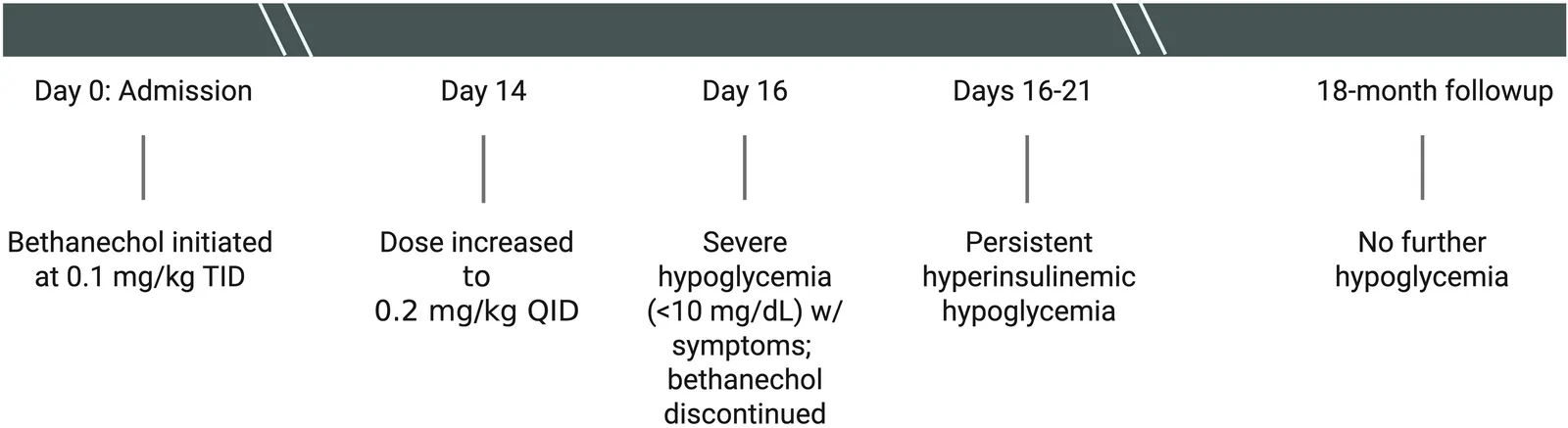
Clinical timeline of bethanechol dosing and hypoglycemia. Abbreviations: TID, three times a day; QID, four times a day. Created in BioRender. Soonavala, R. (2026). https://BioRender.com/4vean39.

Human studies have not demonstrated increased insulin secretion with bethanechol alone, even at high doses [12]. Therefore, direct evidence linking bethanechol to clinically significant HH remains limited. One mechanism is the dependence of muscarinic signaling on extracellular glucose levels. Evidence has demonstrated that acetylcholine and other muscarinic agonists may enhance insulin secretion in response to elevated glucose, but do not provoke insulin release when glucose levels are low [6]. M1 receptors on pancreatic Δ-cells provide inhibitory feedback through somatostatin secretion [13]. These feedback mechanisms may be impaired in autonomic instability. Autonomic instability following spinal cord injury is well documented and may result in poor regulatory responses to hypoglycemia [14]. In this case, increased bethanechol may have amplified an underlying susceptibility to hyperinsulinism in the setting of autonomic instability. This may explain why bethanechol has not been associated with HH in patients without medullary injury.

Although hypoglycemia can occur in children with severe neurologic injury or autonomic instability, it typically follows predictable patterns such as mild hypoglycemia during illness or reduced caloric intake. In this case, the severity, persistence, and insulin-mediated nature were atypical, prompting further evaluation. The temporal association with bethanechol suggests that cholinergic stimulation may have amplified an underlying vulnerability. Medication effects should be considered alongside structural and illness-related causes of hypoglycemia.

A limitation is the persistence of hypoglycemia despite bethanechol's short half-life [15], which may reflect a priming effect or altered pharmacodynamics in the setting of autonomic instability. Gastrointestinal dysmotility may also have prolonged medication absorption. Ultimately, bethanechol may have contributed to the development or persistence of HH in a patient with multiple predisposing factors. Exogenous insulin administration or sulfonylurea exposure was not excluded but was considered unlikely given the inpatient setting, lack of access to these medications, and clinical course.

This case describes a temporal association between bethanechol therapy and HH in a child with severe autonomic instability. Although causality cannot be established, the timing of symptom onset, biochemical findings, and subsequent clinical course raise the possibility that cholinergic stimulation contributed to the observed hyperinsulinism. Clinicians should consider closer glucose monitoring when initiating or escalating cholinergic therapy in medically complex patients.

## Learning points

- Bethanechol may contribute to hyperinsulinemic hypoglycemia in susceptible children with autonomic instability
- Parasympathetic signaling influences insulin secretion and may contribute to dysregulated glucose homeostasis
- Consider glucose monitoring when initiating or escalating cholinergic therapy in medically complex patients

## Contributors

All authors made individual contributions to authorship. R.S. conducted the literature review, contributed to the interpretation of the case and development of the discussion, and created the figures. A.T. was involved in the diagnosis and management of the patient and contributed to the interpretation of the case and underlying pathophysiology. A.K. was involved in the management of the patient, interpretation of the underlying pathophysiology, and conception of the case report. All authors reviewed and approved the final manuscript.

## Data Availability

Original data generated and analyzed for this case report are included in this published article.
